# Mechanism of liver regeneration: 20-year bibliometric analyses

**DOI:** 10.3389/fphar.2023.1190559

**Published:** 2023-06-13

**Authors:** Jingshu Qi, Yunkai Dai, Xin Sun, Chenghai Liu

**Affiliations:** ^1^ Institute of Liver Diseases, Shuguang Hospital Affiliated to Shanghai University of Traditional Chinese Medicine, Shanghai, China; ^2^ Shanghai Key Laboratory of Traditional Chinese Clinical Medicine, Shanghai, China; ^3^ Key Laboratory of Liver and Kidney Diseases, Ministry of Education, Shanghai, China

**Keywords:** mechanism, liver regeneration, CiteSpace, VOSviewer, bibliometric

## Abstract

**Objectives:** The study aims to explore the most influential countries, institutions, journals, authors, “research hotspots,” and trends in the study of the mechanism of liver regeneration (MoLR) in the last 20 years using bibliometric analyses.

**Methods:** The literature associated with the MoLR was retrieved from the Web of Science Core Collection on 11 October 2022. CiteSpace 6.1.R6 (64-bit) and VOSviewer 1.6.18 were used for bibliometric analyses.

**Results:** A total of 18,956 authors from 2,900 institutions in 71 countries/regions published 3,563 studies in different academic journals on the MoLR. The United States was the most influential country. The University of Pittsburgh was the institution from which most articles on the MoLR were published. Cunshuan Xu published the most articles on the MoLR, and George K. Michalopoulos was the most frequently co-cited author. *Hepatology* was the journal in which most articles on the MoLR were published and the most frequently co-cited journal in this field. The research hotspots for the MoLR were origin and subsets of hepatocytes during LR; new factors and pathways in LR regulation; cell therapy for LR; interactions between liver cells in LR; mechanism of the proliferation of residual hepatocytes and trans-differentiation between cells; and prognosis of LR. The emerging topic was the mechanism of regeneration of a severely injured liver.

**Conclusion:** Our bibliometric analyses provide (i) a comprehensive overview of the MoLR; (ii) important clues and ideas for scholars in this field.

## Introduction

“Liver regeneration” (LR) refers to autonomous restoration of the mass and function of a damaged liver. LR includes the induction of cytoprotective mechanisms, deletion of mortally wounded cells, repair of less damaged surviving cells, liver cell proliferation to replace the cells that have died, deposition of new matrix, and tissue remodeling (Diehl, 2002). Of these actions, hepatocyte proliferation is the central event. As a powerful inherent ability of the liver, LR can occur in liver injury caused by partial hepatectomy, acute liver injury induced by chemical agents or viruses, liver fibrosis, etc. (Shang et al., 2016; Clemens et al., 2019; Yagi et al., 2020). The repair of a damaged liver and maintenance of liver homeostasis are extremely important. Moreover, understanding the MoLR can aid the treatment and prognosis of various types of liver diseases.

Research on the MoLR has been relatively deep, but quantitative data are lacking. Although some scholars have used meta-analysis to discuss studies with conflicting data (Asnaashari et al., 2021), the depth of discussion has been relatively limited and subjective. In addition, research on the MoLR by many scholars has, in general, been limited to literature reading and summaries of personal clinical experience.

“Bibliometrics” is a discipline that uses mathematical, statistical, and other quantitative methods to analyze and describe relevant literature. The research content includes not only descriptive statistics (e.g., countries/regions, institutions, journals, authors, keywords, and references) but also network analysis (e.g., links between authors and institutions, “literature clusters”) (Ninkov et al., 2022).

We wished to systematically review and identify the “research hotspots” and development trends in LR. Two visualization tools, VOSviewer and CiteSpace, were used to review and analyze the literature on LR from 2003 to 2022.

## Materials and methods

The data analyzed in this study were retrieved on 11 October 2022 from two influential databases: Web of Science Core Collection and Science Citation Index Expanded.

Then, advanced retrieval was undertaken according to the following search strategy: [TS = (“liver regeneration” OR “hepatic regeneration” OR “hepatocyte proliferation”) AND TS = (mechanism)] OR [TS = (promote OR enhance OR facilitate OR “contribute to”) AND TS = (“liver regeneration” OR “hepatic regeneration” OR “hepatocyte proliferation”)]. The time span was 11 October 2003 to 11 October 2022.

The inclusion criteria for the literature were as follows: (i) the theme of the article was relevant to the MoLR; (ii) the document type was Article or Review; and (iii) the language of the literature was English.

The exclusion criteria for the literature were as follows: (1) Web Of Science categories that were unrelated to the digestive system, medicine, or biology (e.g., respiratory system, chemistry, physics, and polymer science); (2) the theme of the article was not relevant to the MoLR.

The results of retrieval were selected in the form of “Full Record and Cited References” and downloaded in the document format of “Plain Text.”

CiteSpace software (http://cluster.cis.drexel.edu/∼cchen/citespace/) was developed by Professor Chao-Mei Chen (Drexel University, Philadelphia, PA, United States). It can be used to visualize and analyze the scientific literature in a certain field to discover the research hotspots and main research directions in that research area (Chen and Song, 2019). In the present study, annual publication trends were analyzed by CiteSpace 6.1.R6 (64-bit) and visualized by Excel™ 2019 (Microsoft, Redmond, WA, United States). Countries/regions and institutions, co-cited references, “reference bursts” (i.e., the surge in citations of an article within a period of time after publication), and dual-map overlays of journals were analyzed and visualized by CiteSpace 6.1.R6 (64-bit).

The parameters for CiteSpace were set. “Time-slicing” was chosen from November 2003 to December 2022, and the year per slice was 1. Node types were selected once each time. Term source, links, and selection criteria were left as defaults. VOSviewer (www.vosviewer.com/) is a type of bibliometric analytical software developed by Nees Jan van Eck and Ludo Waltman at Leiden University (Leiden, the Netherlands). VOSviewer is employed for mapping knowledge and visualizing keywords and authors (van Eck and Waltman, 2010). In the present study, journals and co-cited journals, authors and co-cited authors, and keywords were analyzed and visualized by VOSviewer 1.6.18.

## Results

**FIGURE 1:**
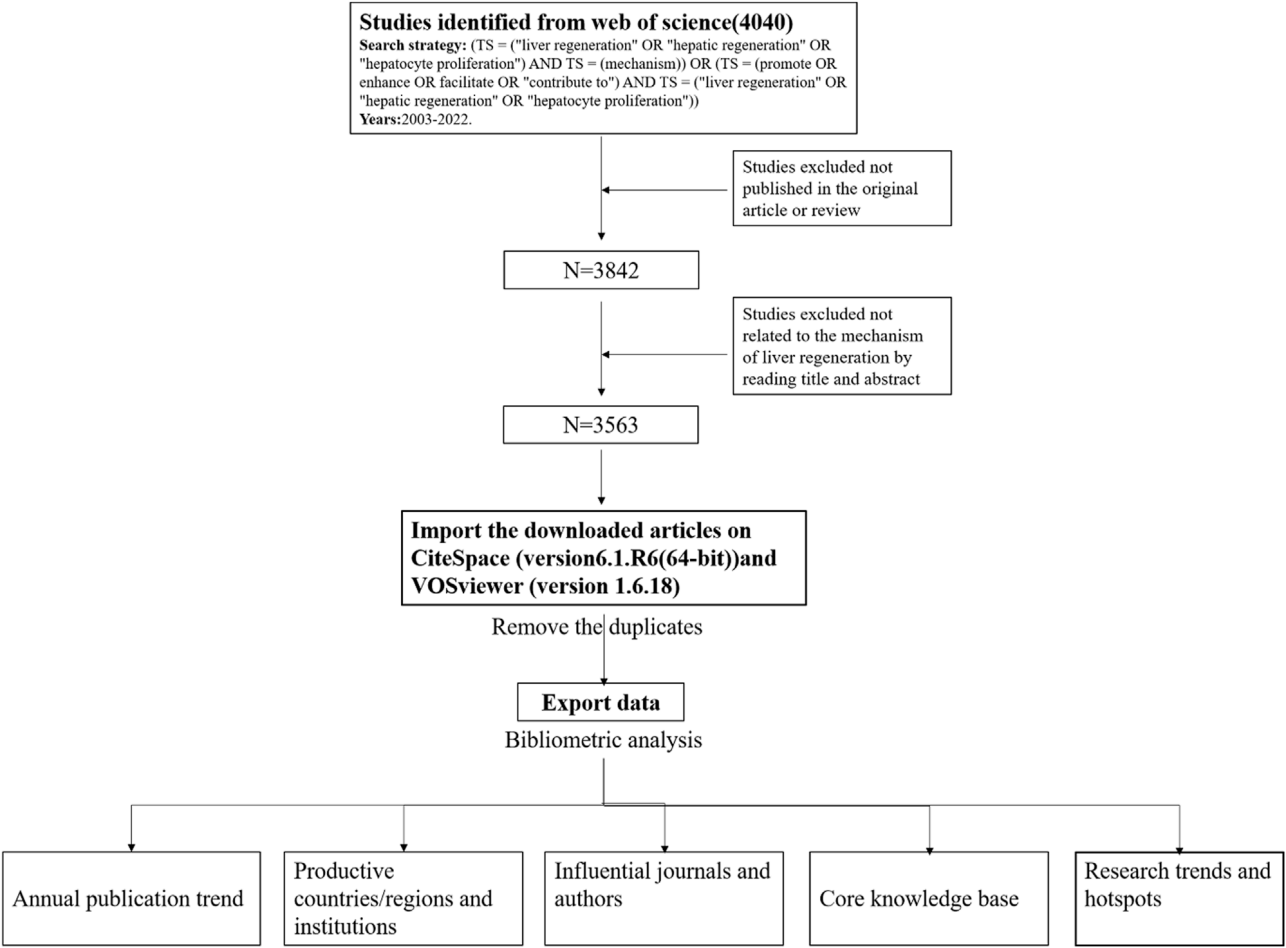
Flowchart of this study.

The flowchart of bibliographic retrieval and research steps in this study are illustrated in Figure 1.

### Annual growth trend of publications

**FIGURE 2:**
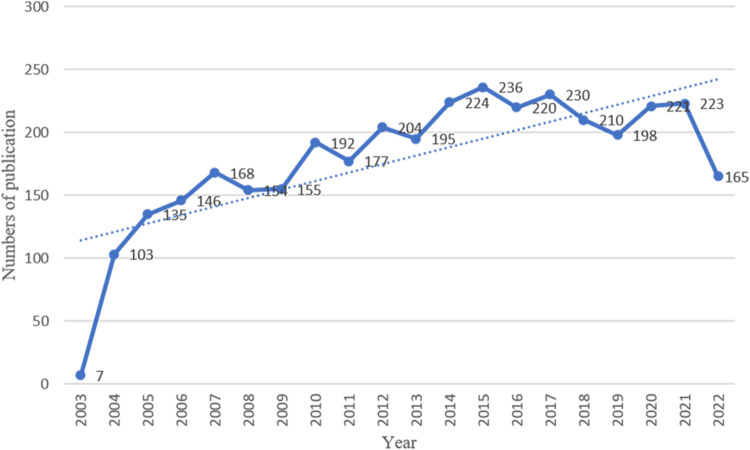
Chronological trend of publications on the mechanism of liver regeneration.

According to the criteria for data selection, 3,563 studies involving the MoLR from 2003 to 2022 were retrieved from Web of Science Core Collection: 3,040 original articles (85.32%) and 523 reviews (14.68%). An overall fluctuating upward trend in the number of articles on the MoLR was observed (Figure 2). The highest number of articles was published in 2015 (236), and the lowest number of articles was published in 2003(7). This observation may have been made because the literature search was started in November 2003.

### Countries/regions and institutions

The 3,563 articles related to the MoLR were written from 2,900 institutions from 71 countries/regions. The United States (*n* = 1,102, accounting for 30.91% of the total) ranked first in the number of published studies, followed by China (*n* = 977, 27.42%), Japan (*n* = 505, 14.17%), Germany (*n* = 342, 9.60%), and Spain (*n* = 151, 4.24%) (Table 1). The United States and China contributed 58.35% of total publications, far more than any other country. China was the only developing country among the top 10 countries. The United States had the highest centrality (i.e., centrality is a parameter used to measure the importance of nodes in a visual graph. The higher the centrality of a node, the closer it is to other nodes and the more important it is. In CiteSpace, nodes with centrality >0.1 are critical nodes.). The other countries/regions with centrality >0.1 were Japan (0.24), Germany (0.17), China (0.16), England (0.15), Spain (0.13), France (0.13), and Switzerland (0.12). Among the top 10 countries/regions in which most articles on the MoLR were published, the United States had 63,702 citations, far more than all other countries, and it also had the highest ratio of citation:publication (57.81), indicating that it published a large number of studies of high quality. China ranked second in the number of citations (18,204), but its ratio of citation:publication was 18.62, the lowest among the 10 countries evaluated.

**TABLE 1:** Top 10 countries/regions and institutions involved studies on the mechanism of liver regeneration.

Rank	Country/region	Article count (%)	Citation	Average citation	Centrality	Institution (country/region)	Article count (%)	Citation	Average citation	Centrality
1	United States	1102 (30.93%)	63702	57.81	0.40	U. Pittsburgh	98 (2.75%)	5130	52.35	0.21
2	China	977 (27.42%)	18204	18.63	0.16	Shanghai Jiao Tong U.	73 (2.05%)	1684	23.07	0.07
3	Japan	505 (14.17%)	15996	31.68	0.24	Zhejiang U.	73 (2.05%)	2759	37.79	0.04
4	Germany	342 (9.60%)	15151	44.30	0.17	Henan Normal U.	65 (1.82%)	449	6.91	0.03
5	Spain	151 (4.24%)	6126	40.57	0.13	U. Tokyo	54 (1.52%)	2989	55.35	0.10
6	France	149 (4.18%)	6134	41.17	0.13	Harvard U.	53 (1.49%)	4791	90.40	0.10
7	Italy	148 (4.15%)	6244	42.19	0.03	U. Kansas	47 (1.32%)	2245	47.77	0.05
8	England	128 (3.59%)	6846	53.48	0.15	Sun Yat Sen U.	47 (1.32%)	1083	23.04	0.04
9	South Korea	99 (2.78%)	2456	24.81	0.06	Capital Med U.	47 (1.32%)	673	14.32	0.06
10	Switzerland	97 (2.72)	4995	51.49	0.12	Chinese Academy of Sciences	45 (1.26%)	1329	29.53	0.11

U., university.

With regard to institutions, the University of Pittsburgh (*n* = 98, accounting for 2.75% of the total) ranked first in the number of published articles, followed by Shanghai Jiao Tong University (*n* = 73, 2.05%), Zhejiang University (*n* = 73, 2.05%), Henan Normal University (*n* = 65, 1.82%), and Tokyo University (*n* = 54, 1.52%). Three-fifths of the top 10 institutions were from China. However, the only institutions with centrality >0.1 were the University of Pittsburgh (0.21) and Chinese Academy of Sciences (0.11). The ratio of citation:publication of Harvard University was 90.4, though it was not in the top five in terms of the number of publications.

**FIGURE 3:**
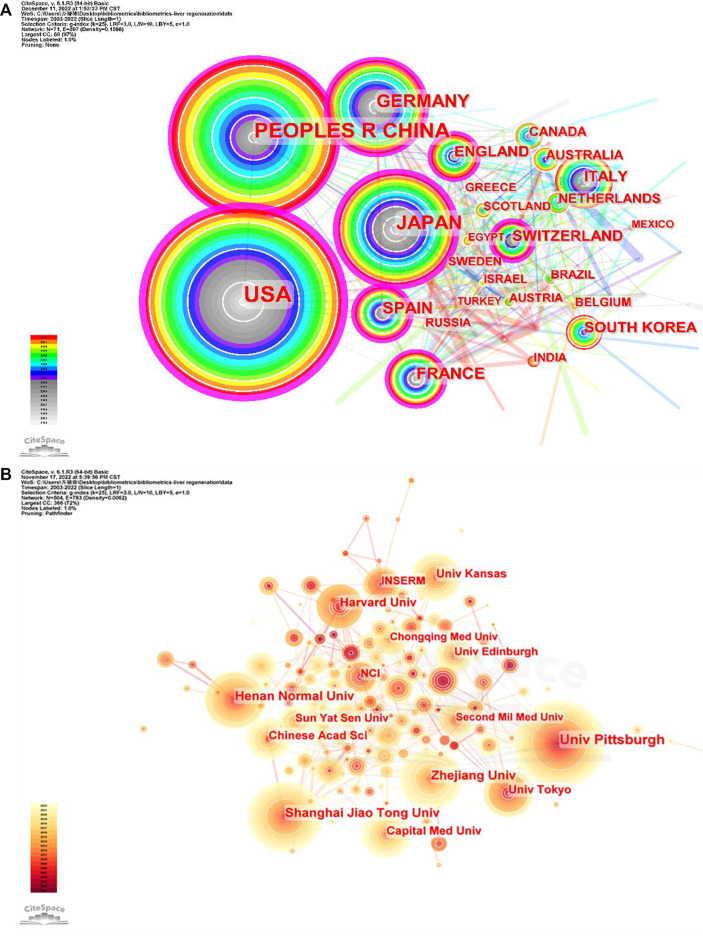
Co-occurrence maps for **(A)** countries and **(B)** institutions. The size of each node represents the co-occurrence frequency and the links reflect the co-occurrence relationships. The color of each node and line indicate different years.

In addition to observing the number of published articles by the size of nodes or finding high-centrality (>0.1) nodes (purple rings in Figures 3A, B), the cooperation between countries/regions or institutions could also be found by the connection between nodes.

### Productive journals and co-cited journals

Statistical analyses suggested that the 3,563 studies were published in 707 academic journals. Table 2 shows the top 10 most prolific and co-cited journals associated with the MoLR. *Hepatology* (234 articles, 6.57%) published the most documents in this field, followed by the *Journal of Hepatology* (*n* = 112, 3.14%), *PLOS One* (*n* = 98, 2.75%), and *World Journal of Gastroenterology* (*n* = 70, 1.96%). In addition, there were three journals in the Q1 Journal Citation Report (JCR) division (i.e., the journal performed better than ≥75% of journals in that category based on its impact factor (IF) score), and the IF of *Gastroenterology* was the highest (33.883).

**TABLE 2:** Top 10 journals and co-cited journals associated with the study of the mechanism of liver regeneration.

Journal	Count	Citation	IF (2022)	JCR (2022)	Co-cited journal	Citation	IF (2022)	JCR (2022)
*Hepatology*	234	15087	17.298	Q1	*Hepatology*	14986	17.298	Q1
*J Hepatol*	112	6101	30.083	Q1	*J Biol Chem*	6400	5.486	Q2
*PLOS One*	98	2969	3.752	Q3	*J Hepatol*	5143	30.083	Q1
*World J Gastroenterol*	70	1191	5.374	Q3	*Proc Natl Acad Sci USA*	4945	12.779	Q1
*J Biol Chem*	63	2990	5.486	Q3	*Gastroenterology*	4814	33.883	Q1
*Sci Rep*	62	1082	4.996	Q3	*Nature*	3933	69.504	Q1
*J Surg Res*	61	1284	2.417	Q3	*Science*	3710	63.714	Q1
*Am J Physiol-Gastr Liver*	58	2021	4.871	Q3	*Cell*	3104	66.85	Q1
*Am J Pathol*	54	2176	5.77	Q2	*J Clin Invest*	3023	19.456	Q1
*Gastroenterology*	52	4770	33.883	Q1	*Am J Pathol*	2592	5.77	Q2

IF, impact factor; JCR, Journal Citation Reports.

With respect to the co-cited journals in Table 2, *Hepatology* (*n* = 14,986) ranked first, followed by the *Journal of Biological Chemistry* (*n* = 6,400), *Journal of Hepatology* (*n* = 5,143)*, Proceedings of the National Academy of Sciences of the United States* (*n* = 4,945), and *Gastroenterology* (*n* = 4,814). In addition, eight journals were located in the Q1 JCR division, and the journal with the highest IF was *Nature* (IF = 69.504).

**FIGURE 4:**
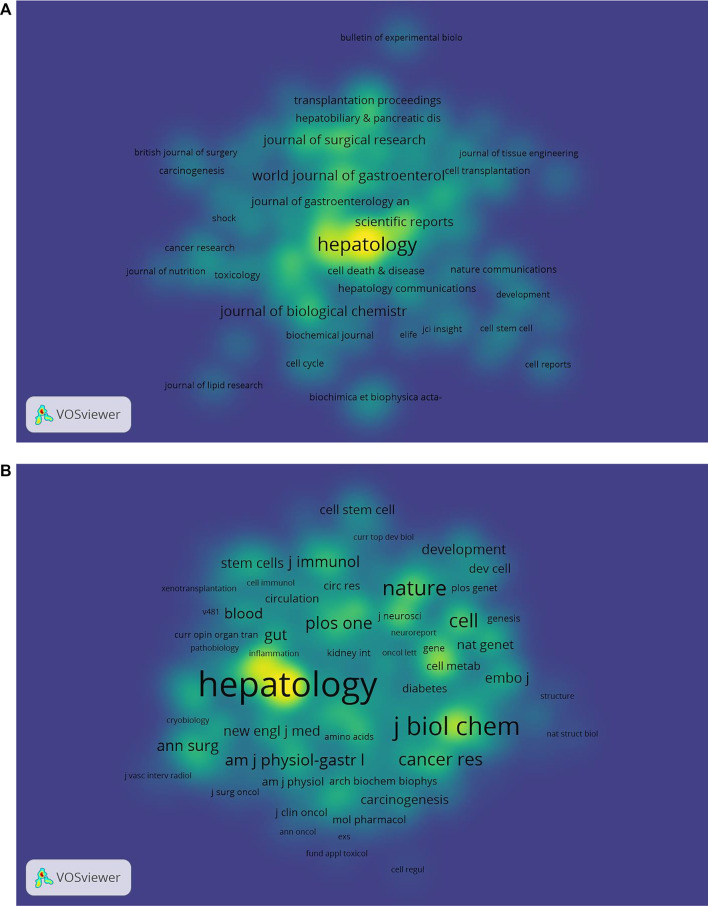
Density maps of **(A)** journals and **(B)** co-cited journals. The size of the word and circle and the opacity of yellow are positively associated with the frequency of co-citation.

Figures 4A, B detail the density views of productive journals and co-cited journals. The density view is particularly useful to obtain an overview of the general structure of a map and to draw attention to the most important areas in a map. Moreover, word size and the shade of yellow were positively correlated with frequency.

**FIGURE 5:**
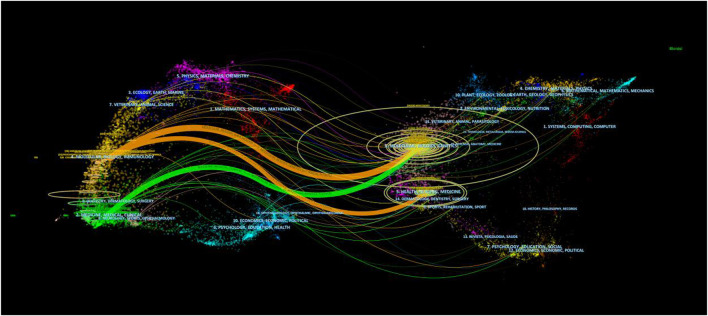
Dual-map overlay of journals associated with the mechanism of liver regeneration. Left: citing journals. Right: cited journals.

The dual-map overlay of journals shows the position of a research subject relative to the main research discipline. Each point on the map represents a journal, and the map is divided into two parts, a citation map on the left and the cited map on the right, and the curves are citation lines. As shown in Figure 5, the mapping identifies three colored primary citation pathways, meaning that research studies published in journals in the field of Molecular/Biology/Genetics and Health/Nursing/Medicine were primarily cited by research studies published in Molecular/Biology/Immunology and Medicine/Medical/Clinical journals.

### Productive authors and co-cited authors

Bibliometric analyses showed that the 3,563 articles were written by 18,956 authors. Cunshuan Xu was top (*n* = 35) in terms of the number of articles published, followed by Wei An (*n* = 24), Udayan Apte (*n* = 23), Dieter Haeussinger (*n* = 23), and Stuart J Forbes (*n* = 22) (Table 3).

**TABLE 3:** Top 10 authors and co-cited authors related to the study of the mechanism of liver regeneration.

Rank	Author	Document	Citation	Co-cited author	Citation
1	Cunshuan Xu	35	304	George K Michalopoulos	1599
2	Wei An	24	343	Nelson Fausto	1258
3	Udayan Apte	23	1187	Rebecca Taub	542
4	Dieter Haeussinger	23	1097	Drew E Cressman	368
5	Stuart J Forbes	22	2140	G M Higgins	454
6	Cuifang Chang	21	829	Yurie Yamada	295
7	Nobuhiro Ohkohchi	21	182	Claudia Mitchell	252
8	George K Michalopoulos	20	1425	Toshikazu Nakamur	281
9	Frank J Gonzalez	20	679	Jaeschke Hartmut	351
10	Satdarshan P S Monga	18	1360	Scott L Friedman	275

According to Price’s Law, the minimum number of papers published by core authors in a certain field (m) is given by

$$m=0.749\times \sqrt{n_{\max}}\;which\;is\approx 5.92,$$

**FIGURE 6:**
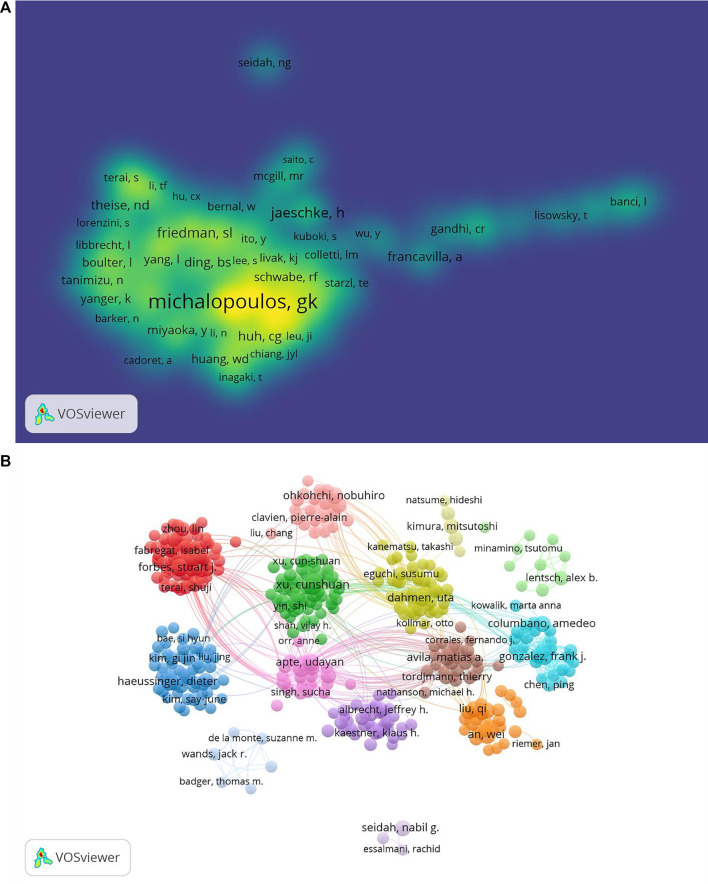
Co-occurrence maps for the mechanism of liver regeneration. **(A)** Authors. **(B)** Co-authors. The size of node indicates the co-occurrence frequency for the author. The different colors reflect different clusters. The links reflect the co-occurrence relationship between authors (map A). The size of the word and circle and the opacity of yellow are positively associated with the co-citation frequency (map B).

$where\;n_{\max}$ represents the number of papers with the most productive authors in the field. Hence, the core authors of the literature written in English in the present study had to write ≥6 publications. Then, 282 core authors were identified through VOSviewer to draw a cooperation network map. As shown in Figure 6A, each node represents an author, and different colors represent different clusters. Authors who collaborated closely are presented in the same color, such as Cunshuan Xu and Shi Yin, Wei An and Qi Liu, Udayan Apte, and Sucha Singh.

“Co-cited authors” are ≥2 authors who are cited simultaneously in ≥1 subsequent article. In our study, 65,625 co-authors were searched. George K Michalopoulos (*n* = 1,599) ranked first, followed by Nelson Fausto (*n* = 1,258), Rebecca Taub (*n* = 542), D. E. Cressman (*n* = 368), and Gregory M. Higgins (*n* = 454) (Table 3). In addition, authors (*n* = 1,248) with co-citations ≥20 were identified to draw a density map (Figure 6B) which could also show the most co-cited authors in LR.

### Keyword co-occurrence and network clusters

**FIGURE 7:**
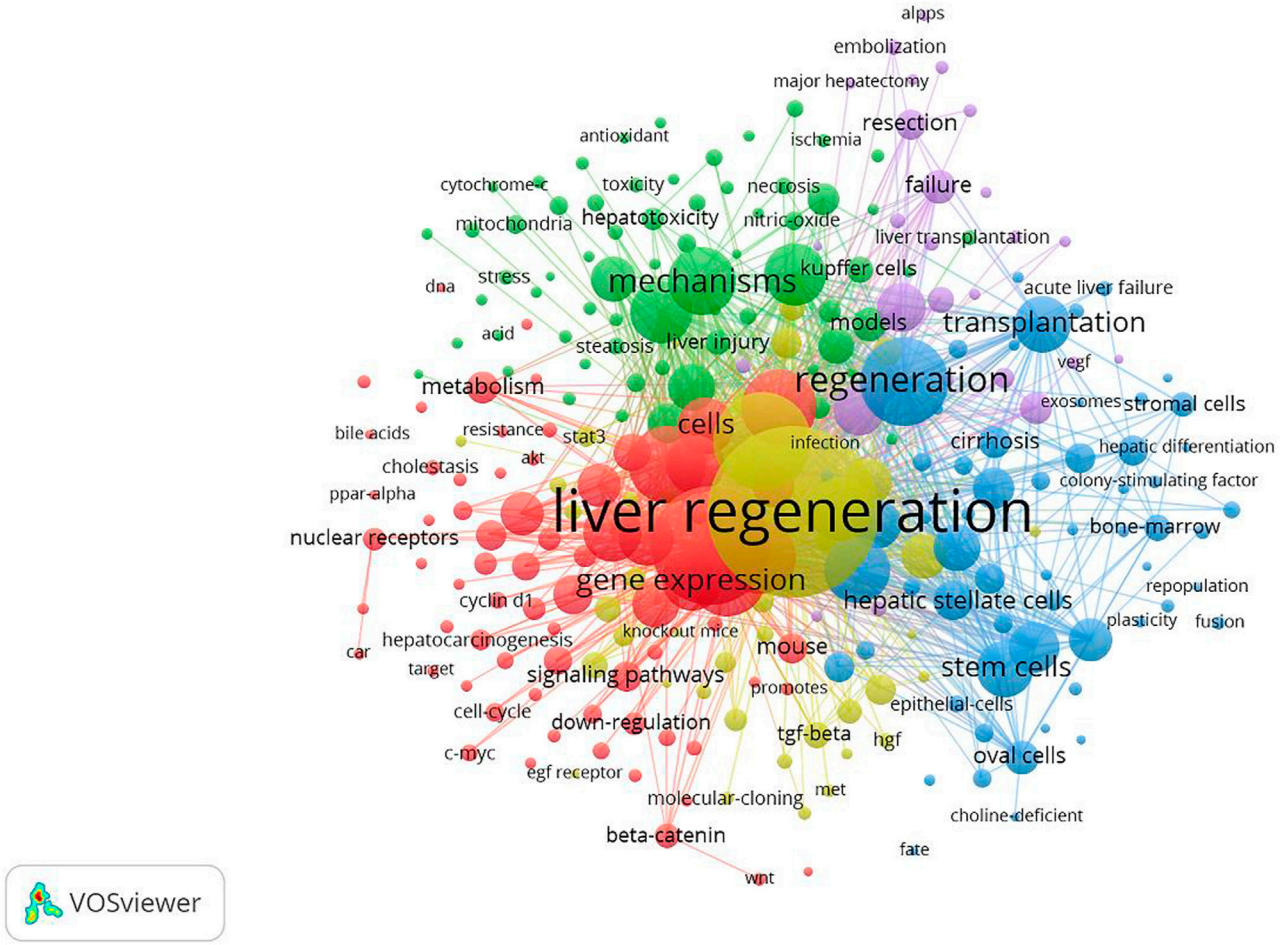
Maps of keywords for the mechanism of liver regeneration and co-occurrence network and clusters. The size of the node and keyword indicates the co-occurrence frequency. The different colors reflect different clusters. The links reflect the co-occurrence relationship.

A total of 718 keywords were obtained from the 3,653 studies through analyses of VOSviewer. In terms of frequency, the keywords “liver regeneration” ranked first (*n* = 1457), followed by “expression” (*n* = 824), “partial hepatectomy” (*n* = 612), “hepatocellular carcinoma” (*n* = 442), “activation” (*n* = 420), “mice” (*n* = 405), “regeneration” (*n* = 403), “mechanism” (*n* = 347), “rat liver” (*n* = 325), and “cell” (*n* = 302) (Table 4), which indicated the research hotspots of the MoLR. Then, a keyword co-occurrence map was established (Figure 7) with the 270 most influential keywords chosen by the criteria of “minimum number of occurrences of a keyword ≥20.”

**TABLE 4:** Top 20 keywords associated with the mechanism of liver regeneration.

Rank	Keyword	Count	Rank	Keyword	Count
1	Liver regeneration	1457	11	Gene expression	301
2	Expression	824	12	Injury	301
3	Partial hepatectomy	612	13	Stem cell	297
4	Hepatocellular carcinoma	442	14	Proliferation	292
5	Activation	420	15	Hepatocyte	284
6	Mice	405	16	Growth	272
7	Regeneration	403	17	Hepatocyte proliferation	257
8	Mechanism	347	18	Transplantation	242
9	Rat liver	325	19	Growth factor	235
10	Cell	302	20	Apoptosis	217

Figure 7 also presents the result of network cluster analyses of keywords. In this map, five clusters represented five research directions and scopes. Among them, cluster 1 (red) was the largest, followed by the clusters 2 (green), 3 (blue), 4 (yellow), and 5 (purple). Specifically, cluster 1 comprised 73 items, including “expression,” “activation,” and “hepatocellular carcinoma.” Cluster 2 contained 66 items, including “mechanism,” “apoptosis,” and “injury.” Cluster 3 consisted of 60 items, including “regeneration,” “transplantation,” and “stem cell.” Cluster 4 comprised 45 items, including “liver regeneration,” “partial hepatectomy,” and “growth factors”. Cluster 5 contained 26 items, including “rats,” “hepatectomy,” and “resection.”

### Co-cited references and burst references

**FIGURE 8:**
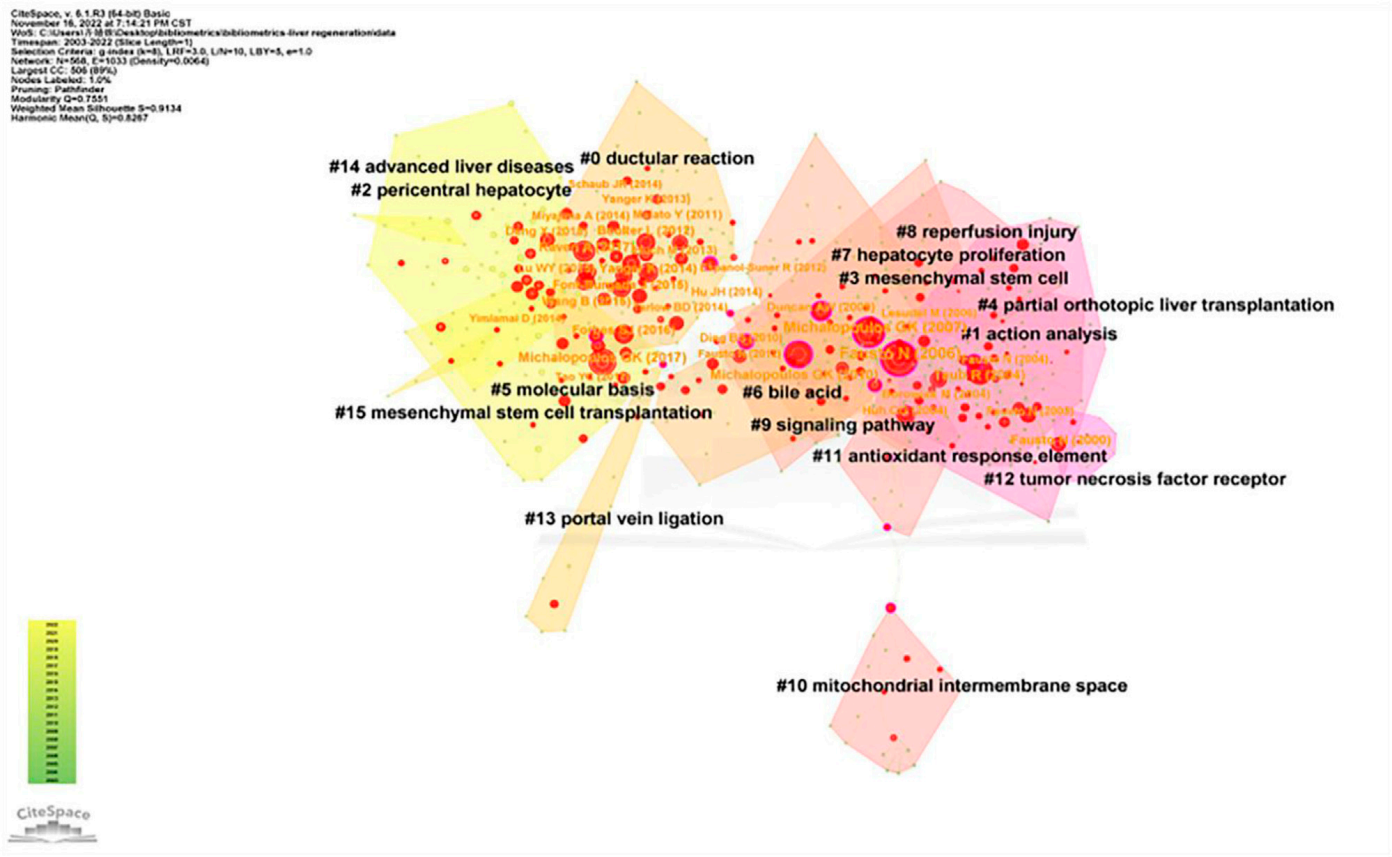
Cluster of co-cited references related to the mechanism of liver regeneration. The different colors represented different clusters. Each point represents a reference. The number on the node represents the cluster to which the reference belongs.

CiteSpace was used for analyses of co-cited references and burst references related to the MoLR. A total of 568 co-cited references on the MoLR in the past 20 years were found through co-occurrence analyses of “co-cited literature” (i.e., two literature works appear together in the citation of a third literature). According to the 10 most frequently co-cited articles (Table 5), “Liver regeneration” by Fausto et al. (2006) (149 co-citations) published in *Hepatology* (IF = 17.298) was the most co-cited article. In addition, the 568 references were classified into 16 clusters through cluster analyses of the co-cited literature in Figure 8 (0 = ductular reaction, 1 = action analysis, 2 = pericentral hepatocyte, 3 = mesenchymal stem cell, 4 = partial orthotopic liver transplantation, 5 = molecular basis, 6 = bile acid, 7 = hepatocyte proliferation, 8 = reperfusion injury, 9 = signaling pathway, 10 = mitochondrial intermembrane space, 11 = antioxidant response element, 12 = tumor necrosis factor receptor, 13 = portal vein ligation, 14 = advanced liver diseases, and 15 = mesenchymal stem cell transplantation). The smaller the cluster number, the greater the influence of that cluster. The top two clusters are shown in Table 6.

**TABLE 5:** Top 10 most frequently co-cited references.

Rank	Citation	Title	First author	Year	Journal
1	149	Liver regeneration	Nelson Fausto	2006	*Hepatology*
2	120	Liver regeneration	George K Michalopoulos	2007	*J Cell Physiol*
3	63	Cholangiocytes act as facultative liver stem cells during impaired hepatocyte regeneration	Alexander Raven	2017	*Nature*
4	53	Adult hepatocytes are generated by self-duplication rather than stem cell differentiation	Kilangsungla Yanger	2014	*Cell Stem Cell*
5	43	Stem cells and liver regeneration	Andrew W Duncan	2009	*Gastroenterology*
6	89	Liver regeneration: from myth to mechanism	Rebecca Taub	2004	*Nat Rev Mol Cell Bio*
7	41	MET provides essential signals for liver regeneration	Malgorzata Borowiak	2004	*Proc Natl Acad Sci USA*
8	25	Combined systemic elimination of MET and epidermal growth factor receptor signaling completely abolishes liver regeneration and leads to liver decompensation	Shirish Paranjpe	2016	*Hepatology*
9	51	Macrophage-derived Wnt opposes Notch signaling to specify hepatic progenitor cell fate in chronic liver disease	Luke Boulter	2012	*Nat Med*
10	25	Distributed hepatocytes expressing telomerase repopulate the liver in homeostasis and injury	Shengda Lin	2018	*Nature*

**TABLE 6:** Top 10 co-cited references associated with the mechanism of liver regeneration.

Rank	Citation	Title	First author	Year		Journal		
A. Top 10 references in cluster 0 “ductular reaction.”								
1	63	Cholangiocytes act as facultative liver stem cells during impaired hepatocyte regeneration	Alexander Raven	2017		*Nature*		
2	53	Adult hepatocytes are generated by self-duplication rather than stem cell differentiation	Kilangsungla Yanger	2014		*Cell Stem Cell*		
3	51	Macrophage-derived Wnt opposes Notch signaling to specify hepatic progenitor cell fate in chronic liver disease	Luke Boulter	2012		*Nat Med*		
4	48	Hybrid periportal hepatocytes regenerate the injured liver without giving rise to cancer	Joan Font-Burgada	2015		*Cell*		
5	47	Self-renewing diploid Axin2 (+) cells fuel homeostatic renewal of the liver	Bruce Wang	2015		*Nature*		
6	45	Hepatic progenitor cells of biliary origin with liver repopulation capacity	Wei-Yu Lu	2015		*Nat Cell Biol*		
7	44	*In vitro* expansion of single Lgr5+ liver stem cells induced by Wnt-driven regeneration	Meritxell Huch	2013		*Nature*		
8	42	Fate tracing of mature hepatocytes in mouse liver homeostasis and regeneration	Yann Malato	2011		*J Clin Invest*		
9	37	Bipotential adult liver progenitors are derived from chronically injured mature hepatocytes	Branden D Tarlow	2014		*Cell Stem Cell*		
10	34	Robust cellular reprogramming occurs spontaneously during liver regeneration	Kilangsungla Yanger	2013		*Gene Dev*		
B. Top 10 references in cluster 1 “action analysis.”								
1	149	Liver regeneration	Nelson Fausto	2006		*Hepatology*		
2	89	Liver regeneration: from myth to mechanism	Rebecca Taub	2004		*Nat Rev Mol Cell Bio*		
3	41	MET provides essential signals for liver regeneration	Malgorzata Borowiak	2004		*Proc Natl Acad Sci USA*		
4	37	Liver regeneration and repair: hepatocytes, progenitor cells, and stem cells	Fausto Nelson	2004		*Hepatology*		
5	23	Liver regeneration	Leonidas G Koniaris	2003		*J Am Coll Surgeons*		
6	20	Liver regeneration	George K Michalopoulos	2005		*Adv Biochem Eng Biotechnol*		
7	18	c-Jun-N-terminal kinase drives cyclin D1 expression and proliferation during liver regeneration	Robert F Schwabe	2003		*Hepatology*		
8	15	The proinflammatory mediators C3a and C5a are essential for liver regeneration	Christoph W Strey	2003		*J Exp Med*		
9	13	Amphiregulin: an early trigger of liver regeneration in mice	Carmen Berasain	2005		*Gastroenterology*		
10	13	Heparin-binding epidermal growth factor-like growth factor links hepatocyte priming with cell cycle progression during liver regeneration	Claudia Mitchell	2005		*J Biol Chem*		

**FIGURE 9:**
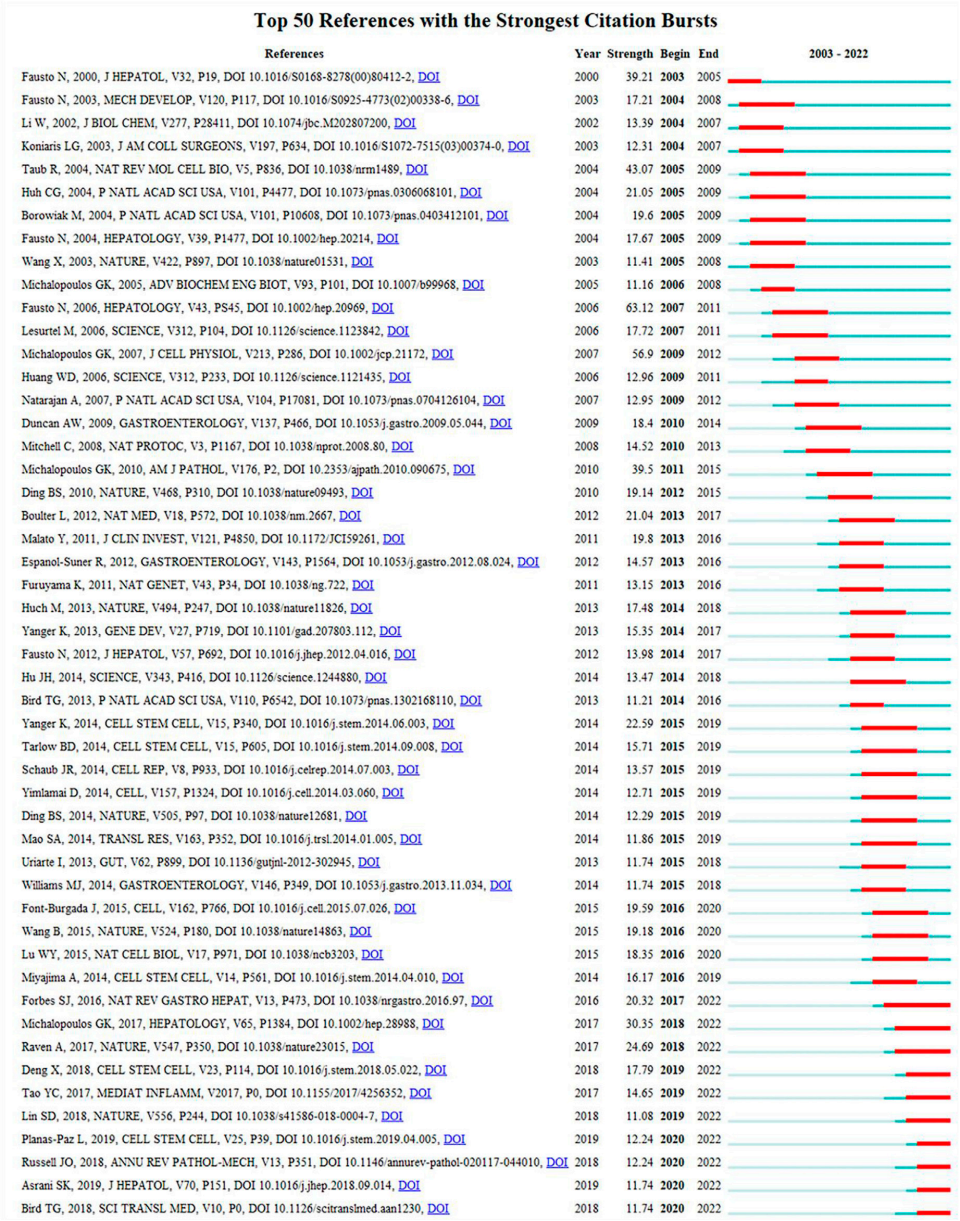
History of burst references. The citation bars denote that the reference has been published. Red bars denote the strength of the burst reference.

Reference burst refers to the surge in citations of an article within a period of time after publication, indicating that scholars have paid great attention to the relevant topic. In this study, 191 burst references were found with the selection criteria in which the minimum duration is ≥2, and the top 50 references are shown in Figure 9. With regard to the strongest burst reference, a review entitled “Liver regeneration” with a strength of 63.12 was published in *Hepatology* by Fausto et al. (2006). In addition, 10 references were considered to still be burst references in 2022.

## Discussion

### General information

The 3,563 articles analyzed in our study were published in 707 journals by 18,956 authors from 2900 institutions from 71 countries/regions.

The number of publications every year suggests that the MoLR received considerable attention in the past two decades, but it also illustrates that there are unsolved problems in this field.

Analyses of countries/regions and institutions focused mainly on three indicators: the number of publications, centrality, and average citation. These indicators can reflect the influence of a country/region and institution in a research field. In the present study, the country with the highest number of publications on LR was the United States (*n* = 1102), followed by China (*n* = 977) and Japan (*n* = 505) (Table 1). Moreover, the United States had the highest centrality (0.4), indicating that it played an important part as a “bridge” in cooperation between countries. In addition, the institution with the most publications on LR was the University of Pittsburgh (*n* = 98), followed by Shanghai Jiao Tong University (*n* = 73) and Zhejiang University (*n* = 73). The countries and institutions with the highest average citation were the United States (article count/citation = 57.81) and Harvard University (article count/citation = 90.40). In terms of the number of articles published on LR, China ranked second in the world, but its average citation was <20, which may have been caused by the immaturity of science and technology infrastructure in China. The links between countries/regions and institutions shown in Figure 3 can reflect their cooperative relationships, but obvious patterns were not found in our study.

*Hepatology* was the journal in which most articles on LR were published (*n* = 234) and was the most frequently co-cited journal (*n* = 14,986), which indicated its influential status in study on the MoLR. Furthermore, the dual-map overlay of journals suggested that research on the MoLR was mainly focused on basic research and translational medicine.

Cunshuan Xu was the most frequently published author (*n* = 35) and George K Michalopoulos had the most co-citations (*n* = 1599) on LR, indicating they had a potential outstanding contribution to the study of LR (Table 3; Figure 6).

### Core knowledge base

Co-cited literature means that two literature works form a co-cited relationship, which characterizes the core knowledge base of a field. The cited literature in the present study was divided into 16 clusters. The top two clusters with the highest influence were labeled as “Part A” and “Part B.”

#### Part A

Part A was clustered as a “ductular reaction” (DR). The DR is a repair response of damage to hepatocytes and bile duct cells. The DR was first proposed by Hans Popper in the United States and later defined as “a reaction of the ductular phenotype, possibly but not necessarily of ductular origin” (Popper et al., 1957; Roskams et al., 2004), that is, an increase in cytokeratin-19^+^ cells. However, in addition to cholangiocyte proliferation, the origin of these cells can be derived from activated hepatic progenitor cells (HPCs) or hepatocytes. Apart from bile duct hyperplasia, a bile duct reaction also shows infiltration by inflammatory cells in portal areas, angiogenesis, and activated HPCs (Sato et al., 2019). The contents of the top ten studies in this cluster included four main aspects, as discussed in the following section.

##### Source of hepatocytes in LR

Four articles addressed the issue of hepatocyte origin during liver homeostasis as well as acute and chronic liver injuries. Using two-thirds partial hepatectomy and an acute intoxication model based on carbon tetrachloride (CCl_4_) in mice, Malato et al. (2011) suggested that new hepatocytes generated during liver homeostasis and acute hepatocyte injury originated from preexisting hepatocytes. Yanger et al. (2014) concluded that hepatocytes were the main source of hepatocyte renewal/regeneration in the adult liver regardless of the type of injury. They employed a 3,5-diethoxycarbonyl-1,4-dihydrocollidine (DDC) and choline-deficient ethionine-supplemented diet, CCL_4_, and an α-naphthyl-isothiocyanate diet in mice. Wang et al. (2015) identified a subset of hepatocytes that maintained the homeostasis of hepatocyte self-renewal in the normal liver: axis inhibition protein 2^+^ hepatocytes. Font-Burgada et al. (2015) discovered hybrid periportal hepatocytes, which replenish hepatocytes after their chronic depletion. Raven et al. (2017) demonstrated that bile duct cells can act as facultative liver progenitor cells in a model using DDC, methionine- and choline-deficient diet combined with thioacetamide, and CCL_4_-induced liver injury in tandem with inhibition of hepatocyte proliferation.

##### Source of bile duct cells in LR

Yanger et al. (2013) found that in the absence/dysfunction of biliary epithelial cells (BECs) in the DDC model and bile duct ligation model in mice, hepatocytes could transform into bile duct epithelial cells. This process required intervention by the Notch signaling pathway. The results obtained by Malato et al. (2011) through bile duct ligation and DDC model argued against the idea that biliary duct injury leads to the conversion of hepatocytes into BECs. Huch et al. (2013) found that liver damage-induced leucine-rich repeat-containing G-protein-coupled receptor 5 liver stem cells could generate hepatocytes and cholangiocytes *in vivo* and could be cloned and expanded into organoids and could differentiate into hepatocytes *in vitro*.

##### Source of HPCs in LR

In addition to being investigated as a source of hepatocytes/bile duct cells (Lu et al., 2015), the origin of HPCs has also been discussed. Tarlow et al. (2014) used a DDC model in mice. Hepatocytes could be converted to a unique progenitor state that could be reversed upon recovery and, therefore, be the source of HPCs in chronic liver injury.

##### Mechanism of transformation of HPCs into bile duct cells or hepatocytes

During biliary regeneration, myofibroblasts express Jagged-1, which promotes Notch signaling in HPCs and converts HPCs into bile duct cells. During hepatocyte regeneration, macrophage phagocytosis of hepatocyte debris induces wingless-type (Wnt)3a expression. This action activates the Wnt signaling pathway in nearby HPCs, thereby promoting Numb expression in HPCs and converting HPCs to hepatocytes (Boulter et al., 2012).

In summary, the issue of various liver cell sources in LR is controversial. Accumulating evidence suggests that the origin of regenerated hepatocytes is dependent upon the animal model selected and experimental conditions. Moreover, the proliferation of residual hepatocytes and trans-differentiation between cells are important features of LR.

#### Part B

Part B was clustered as action analyses, which can be understood as mechanism analyses. Five reviews and five articles were in the top 10 literature works, all of which discussed the MoLR. The related contents are described in the following section.

##### Cytokines, growth factors, cytokine/growth factor-mediated pathways, and other regulatory factors in LR

Koniaris et al. (2003) summarized the regulatory factors and intracellular pathways in the three stages of LR: initiation, proliferation, and termination. The regulatory factors included tumor necrosis factor (TNF) and interleukin-6 in the initiation phase; hepatocyte growth factor (HGF), transforming growth factor (TGF)-α, epidermal growth factor, insulin, and epinephrine in the proliferation phase; and activin A, TGF-β, and latency-associated peptide and follistatin in the termination phase. Moreover, the intracellular pathways involved were signaling by the TNF receptor and nitric oxide in the initiation phase; signaling by G-protein-coupled receptors, receptors with intrinsic tyrosine kinase activity, receptors activating the Janus kinase/signal transducer and activator of transcription pathway, and steroid and thyroid hormone receptors in the proliferation phase; and serine and threonine kinase receptors for the TGF-superfamily in the termination phase. Then, Taub (2004) attempted to define regions of overlap between cytokine/growth factor-mediated pathways. For example, the downstream targets that were shared by the two pathways included activator protein 1, Jun amino-terminal kinase, phosphorylated extracellular signal-regulated kinases, CCAAT-enhancer-binding protein-β, and insulin-like growth factor-binding protein 1. Furthermore, five articles (Schwabe et al., 2003; Strey et al., 2003; Borowiak et al., 2004; Berasain et al., 2005; Mitchell et al., 2005) discussed other regulatory factors in LR. These included proteins that trigger LR initiation (e.g., Jun amino-terminal kinase, C3a/C5a, and amphiregulin) and key factors in the proliferative phase of hepatocytes (e.g., mesenchymal–epithelial transition factor and heparin-binding epidermal growth factor).

##### Cellular mechanisms of LR focusing on the role of hepatocytes, oval cells, or bone marrow cells as the cellular source of LR

As stated in the review by Fausto (2004), in general, replication of existing hepatocytes was the quickest and most efficient way to generate hepatocytes for LR and liver repair. Oval cells replicated and differentiated into hepatocytes only if the replication of mature hepatocytes was delayed or blocked entirely. Bone marrow cells could generate hepatocytes in transplanted livers, but the number of hepatocytes produced was very low. However, bone marrow cells were an important source of nonparenchymal (e.g., Kupffer and endothelial) cells.

##### Interactions between liver cells in LR

The review written by Michalopoulos and DeFrances (2005) was only one of five reviews that proposed interactions between liver cells in LR. Hepatocytes can produce various growth-regulating cytokines (e.g., TGFα, vascular endothelial growth factor (VEGF), fibroblast growth factor (FGF)-1, FGF-2, angiopoietin-1, angiopoietin-2, and heparin-binding epidermal growth factor) which stimulate the growth of adjacent (e.g., endothelial, stellate, and bile duct epithelial) cells. However, growth factors affecting hepatocytes (e.g., HGF) can also be produced by neighboring stellate cells and endothelial cells. In addition, VEGF produced by replicating hepatocytes can stimulate endothelial cells to produce the hepatocyte mitogen HGF via VEGF receptor 1.

##### Role of liver metabolism in LR

Fausto et al. (2006) stated that LR was not only related to cytokines and growth factors but also to liver metabolism. Amino acids regulate hepatocyte proliferation by controlling the expression of cyclin D1. After partial hepatectomy, the activity of p70 S6 kinase increases, and the activity of eukaryotic cell initiation factor 4E-binding protein 1 (translational repressor) decreases, leading to increased translation.

In summary, research on cytokines, growth factors, and their related pathways in LR is detailed, and the discovery of new regulatory factors is a research trend. The related content is not comprehensive, but the topic of interactions between liver cells in LR is a research hotspot because most regulatory factors are secreted by cells.

##### Analyses of hotspots and emerging topics

Analyses of keywords can provide a glimpse of the topic of an article. The keyword co-occurrence can reflect the research hotspots and trends of a subject in a certain field. The top 20 keywords with high-frequency terms involved in the MoLR are shown in Table 4. We can summarize (approximately) five main areas in this field through the keywords shown in Table 4. The first area is the commonly used model of LR, which was partial hepatectomy in rats/mice. Second, the main source of hepatocytes during LR after acute or chronic liver injury was the proliferation of residual hepatocytes. However, if the liver was severely damaged or hepatocyte proliferation was inhibited, liver stem cells (e.g., liver progenitor cells) could be used as the hepatocyte source. Third, the only treatment for end-stage liver disease was liver transplantation. However, due to the shortage of organs, the practical application of liver transplantation was limited. Primary-cell transplantation is expected to become an alternative method to organ transplantation. Fourth, HGF is a key factor in LR promotion. Fifth, the adverse consequences of LR are the cytokines produced during LR, which can induce the recurrence and metastasis of hepatocellular carcinoma.

Clustering analyses of keywords provided a more intuitive view of the research hotspots in the MoLR, as represented by five clusters (red, green, blue, yellow, and purple) in Figure 7. Cluster 1 focused on the targets that regulated LR. Cluster 2 involved the etiology of liver injury, chronic diseases, and the pathological basis of liver injury. Cluster 3 centered on cell therapies to promote LR. Cluster 4 mainly covered the factors and pathways that regulate LR. Cluster 5 mainly involved the commonly used models of LR, as well as angiogenesis in LR.

Burst references can reflect the emerging research topics in a certain field. Herein, 191 burst references were noted, and the top 50 are shown in Figure 9. Based on the strength of burst references (high to low), 10 references could be focused upon.

The first paper (Michalopoulos, 2017) (strength = 30.35) was a review by George K. Michalopoulos published in *Hepatology*, which summarized the MoLR after partial hepatectomy. The second paper (Raven et al., 2017) (strength = 24.69) was by Alexander Raven et al. published in *Nature* and discussed the knowledge base of LR. The third paper (Forbes and Newsome, 2016) (strength = 20.32) was by Stuart J. Forbes and Philip N. Newsome and published in *Nature Reviews Gastroenterology and Hepatology*. They summarized the animal models used and MoLR (including factors, signaling pathways, and interaction between hepatic cells). They also pointed out that the regeneration mechanism of a severely injured liver is an important question that warrants investigation. The fourth paper (Deng et al., 2018) (strength = 17.79) was written by Xing Deng and colleagues and published in *Cell Stem Cell*. The results showed that BECs are converted into hepatocytes through a hepatocyte nuclear factor-4α^+^ cytokeratin-19^+^ bi-phenotypic state in severe liver injuries. The fifth paper (Tao et al., 2017) (strength = 14.65) was by Yachao Tao et al. and published in *Mediators of Inflammation*. They reviewed the novel and important signaling molecules involved in LR. The sixth paper (Planas-Paz et al., 2019) (strength = 12.44) was by Lara Planas-Paz and coworkers and published in *Cell Stem Cell*. They demonstrated that signaling by Yes-associated protein and mammalian target of rapamycin complex 1 promoted BEC expansion during DR. G-protein-coupled receptors 4/5-mediated Wnt/β-catenin signaling is dispensable for promoting DR. The seventh paper (Russell and Monga, 2018) (strength = 12.44) was by Jacquelyn O. Russell and Satdarshan P. Monga and published in *Annual Review of Pathology: Mechanisms of Disease*. That article mainly discussed the Wnt/β-catenin signaling pathway, its role in cell–cell adhesion and liver function, and the cell type-specific roles of the Wnt/β-catenin signaling pathway in liver physiology/disease. The eighth paper (Asrani et al., 2019) (strength = 11.74) was a review written by Sumeet K. Asrani and colleagues and published in the *Journal of Hepatology*. They discussed the global epidemiology and prognosis of various acute and chronic liver diseases. The ninth paper (Bird et al., 2018) (strength = 11.74) was by Thomas G. Bird and colleagues and published in *Science Translational Medicine*. They found that severe acute hepatic necrosis induced the spread of senescence to remaining viable hepatocytes, which impaired hepatocyte-mediated regeneration. Furthermore, the spread of senescence was dependent upon a macrophage-derived TGF-β1 ligand. The tenth paper (Lin et al., 2018) (strength = 11.08) was by Shengda Lin and coworkers and published in *Nature*. They found that hepatocytes with high telomerase expression regenerated hepatocytes and replenished the liver parenchyma during liver homeostasis and liver injury.

### Study limitations

The present study had three main shortcomings. First, the literature was obtained from the Web of Science Core Collection. Although this database is representative, some relevant literature may have been overlooked. Second, the articles whose topics were unrelated to the MoLR were excluded manually, but some relevant literature may have been overlooked. In the section on the core knowledge base, we discussed the literature only in the first two clusters.

## Conclusion

The MoLR remains a research hotspot worthy of investigation. Our study elicited four main findings. First, the most influential country in study of the MoLR was the United States. It had the highest number of published papers, centrality, and citation:publication ratio. Second, the University of Pittsburgh ranked first in the number of published papers on LR and centrality. Second, *Hepatology* was the journal in which most articles on LR were published and the most frequently co-cited journal, which indicated its influential status in the study of the MoLR. Third, the most productive author in LR was Cunshuan Xu, and the top co-cited author was George K Michalopoulos. Fourth, the research hotspots related to MoLR were the origin and subsets of hepatocytes during LR; new factors and pathways in LR regulation; cell therapy for LR; interactions between liver cells in LR; mechanism of the proliferation of residual hepatocytes and the trans-differentiation between cells; and prognosis of LR. Fifth, the emerging topic to which attention should be paid is the regeneration mechanism of a severely injured liver.

## Data Availability

The original contributions presented in the study are included in the article/Supplementary Material; further inquiries can be directed to the corresponding author.
